# Climate change as observed through the IMS radionuclide station in Spitzbergen

**DOI:** 10.1038/s41598-024-59319-6

**Published:** 2024-05-13

**Authors:** Jolanta Kuśmierczyk-Michulec, Jonathan Baré

**Affiliations:** International Data Centre, Comprehensive Nuclear-Test-Ban Treaty Organization, PO Box 1200, 1400 Vienna, Austria

**Keywords:** Climate sciences, Environmental sciences

## Abstract

The International Monitoring System (IMS), installed and maintained by the Comprehensive Nuclear-Test-Ban Treaty Organization (CTBTO) with the support of States Signatories, is a global system of monitoring stations based on four complementary technologies: seismic, hydroacoustic, infrasound and radionuclide. One of the IMS radionuclide stations is located in Spitzbergen, the largest island of the Norwegian Svalbard Archipelago, which borders the Barents Sea and the Northern Atlantic Ocean. It has been demonstrated that signs of climate change are particularly noticeable in that region. As many other radionuclides observed in environmental measurements, ^212^Pb is always observed at IMS stations, in varying quantities. This is also the case for the IMS station RN49, Spitzbergen, where it can be demonstrated that the average concentration of the measured lead ^212^Pb increases. This is observable specifically October through December. This paper demonstrates the asset of IMS data to study climate change effects. Our conclusions are supported by global temperature anomaly data from NOAA’s Global Surface Temperature Analysis, covering the period 1850 to 2023.

## Introduction

The International Monitoring System (IMS), installed and maintained by the Comprehensive Nuclear-Test-Ban Treaty Organization (CTBTO), is a unique global system of monitoring stations that, when complete, will consist of 321 monitoring stations and 16 laboratories hosted by 89 countries around the globe. The IMS uses four complementary verification methods, based on the latest available technologies. They are permanently monitoring the globe, searching for evidence of nuclear explosions, providing a steady flow of (near) real-time data.

**Figure 1:**
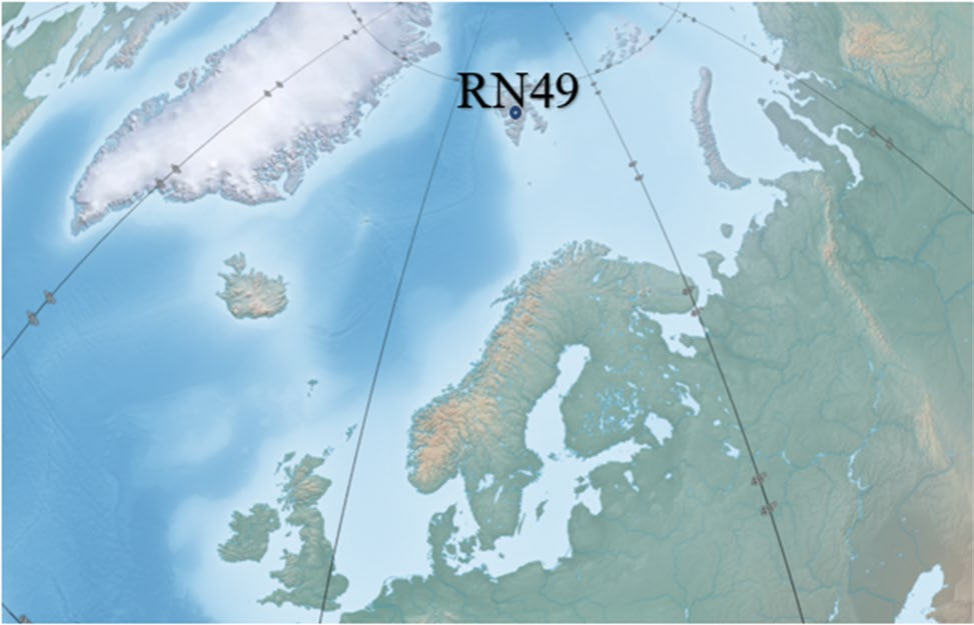
Map showing the location of IMS station RN49 in Spitzbergen. Made with Natural Earth (https://www.naturalearthdata.com/).

One of the IMS radionuclide stations is operated in Spitzbergen (see Fig. 1), the largest island of the Svalbard archipelago in northern Norway, which borders the Barents Sea and the Northern Atlantic Ocean. Due to its specific location (about midway between the northen coast of Norway and the North Pole) and based on many lines of evidence, the signs of global warming have resulted in noticeable climatic changes on Svalbard^1^.

**Figure 2:**
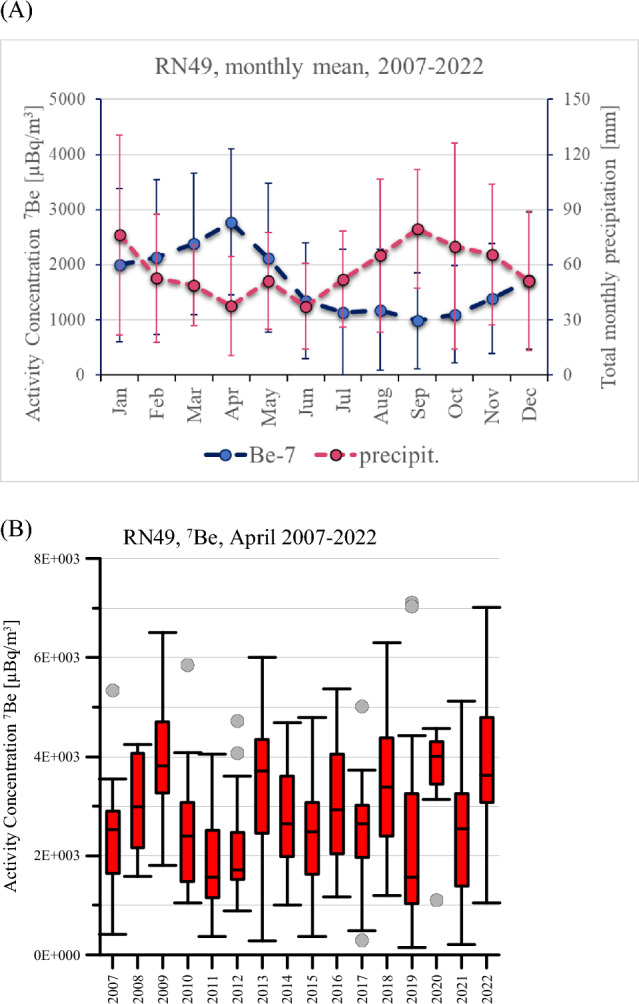
Measurements of ^7^Be collected at the station RN49. (**A**) Relation between the activity concentration of ^7^Be (blue dots) and the precipitation level (red dots). (**B**) Yearly variations in the ^7^Be monthly values.

The Arctic region is particularly vulnerable to climate change because the global warming has been much faster there than in the rest of the world. This phenomenon is known as Arctic amplification^2,3^. A recent estimation has revealed that during the last 43 years the Arctic has been warming nearly four times faster than the rest of the globe^4^, which is a higher ratio than generally reported in literature.

Warming of the atmosphere and oceans has been accompanied by a strong decline in the Arctic Sea ice coverage, impacting the Arctic Sea ice thickness from one hand^5–9^ and triggering sea level rise^10^ on the other hand. With the increased number of satellites and development of satellite imagery, it was possible to detect changes in the Arctic Sea ice extent, a pronounced feature of Arctic climate change. Willmes^11^, using a 20-year climatology from satellite observations for wintertime (November to April), have demonstrated that the weakening of sea ice and the occurrence of sea-ice leads (where lead is defined as a fracture through sea ice that can be used for navigation purposes^12^) is influenced by the ocean bathymetry and associated currents. The authors have identified a number of lead “hot spots”, for example in Fram Strait, in the Barents and Kara Seas but also northwest of Svalbard. They also demonstrated that a generally higher monthly lead fraction is observed in November, i.e. at the beginning of the winter, than in April.

The relationship between dominant spatial features of surface wind variability and the Arctic Sea ice extent has been investigated by many researchers^13,14^. Wu^15^ by applying the Complex Vector EOF (CVEOF) method to the monthly mean sea level pressure and surface wind fields representing summer season (July to September), for the period 1979–2010, found that September sea ice extent minima are mainly associated with the negative phase of the Arctic Dipole pattern and the positive phase of the Central Arctic pattern. In addition, both phases coherently showed an anomalous anticyclone over the Arctic Ocean^15^.

Observations made by different technologies provide a more complete picture of all modifications related to climate change. For example, satellite observations allowed registration of unprecedented quantities of heat entering the Pacific sector of the Arctic Ocean through the Bering Strait during summer months of 2018^16^. Such meandering warm jets offshore of the Barrow Canyon are not unusual^17^, what may lead to accelerating sea ice melt in the region^16^. Hydroacoustic observations allow to register underwater noise produced by tiny air bubbles released from melting glacier ice^18^. A direct link between the melt rate and related noise, facilitates a study of subsurface melting in a direct way.

In this study, we aim at demonstrating that the global monitoring network of the CTBTO can also be used to indicate climate change by adopting a new perspective on the data collected by the radionuclide component of the IMS system. For that purpose, we focus on ^212^Pb, a radionuclide of terrestrial origin observed throughout the IMS network in varying concentrations and quantified during the analysis of spectra. In routine analysis, the activity concentration of ^212^Pb serves as an indicator (among others) for the measurement system performance. ^212^Pb may also be connected to climate variations. For example, the increase in surface temperature directly influences the activity concentration of ^212^Pb released from the Earth surface and indirectly shows trends in climate variations. For more than 20 years, starting in 2003, ^212^Pb has been measured daily by the CTBTO at all stations of the IMS network, and quantified. Revisiting this large amount of operational data and interpreting it from a different perspective may bring another evidence of climate change.

To complement the radionuclide observations, atmospheric transport modelling results were used. Atmospheric backward simulations for the station RN49 produced operationally on a daily basis, for the period from 2008 to 2022, were used to trace air masses back to their possible origin. This was done to investigate how often air masses arriving at RN49 were coming from the dust sources (e.g. in Alaska, Canada, Denmark, Greenland, Iceland, Svalbard, Sweden and Russia) recently identified^19^. Dust particles from these regions represent soil particles and differ from the crustal dust of the Sahara. For example, Icelandic dust is of volcanic desert origin, rather dark with high proportion of heavy metals^19^. Dust and organic matter (e.g. ice algae) deposited on snow and ice, can reduce surface albedo and accelerate the melting of glaciers^20,21^. Therefore, identification of potential sources of air masses arriving at RN49 can provide additional insights of possible contributing factors to change in surface temperature.

The next two sections provide more information on radionuclide measurements used in this study (Sect. 2), and on atmospheric transport modelling (Sect. 3). Section 4 addresses the question whether natural radionuclides can be used as tracers of climate change. Section 5 includes a discussion on potential sources of air masses arriving at the RN49 station, in the context of climate change.

## Experimental data

The measurement of activity concentrations of radioactive particulates is directly performed at all IMS stations. Several technologies are used, but their general operating principles are very similar. A volume of air is continuously collected with a 500–1000 m^3^/h air flow and forced to pass through filter papers to retain particles. The particulate radionuclides collection efficiency is good, as the collectors retain more than 85% of particles with diameter larger than 0.2 μm. Each sample is collected during a 24 h period, and then cooled for 24 h to let all short-lived radionuclides that are not of interest for the CTBTO decaying away (reducing at the same time the detection limits). The activity concentration of aerosol-bound radionuclides is then measured with high-resolution germanium (HPGe) detectors^22,23^.

In this study, the radionuclide station RN49 is considered. The station is located at Platåberget, Spitzbergen, Norway (coordinates 78.2N, 15.4E), at an elevation of 460 m above sea level. Platåberget is a plateau mountain with a large variety of surface forms, ranging from pebbles to blocks of 1 m in diameter. The station started sending data to the PTS on the 21st of July 2002, and was certified after a certification visit in early October 2002.

Meteorological data used in this study was collected by meteorological equipment placed at the sampling stations and recorded every 10 min. The recorded parameters include air temperature, wind speed, wind direction, relative air humidity, atmospheric pressure and rainfall. Data from the meteorological station were averaged over 24 h. The meteorological data together with collocated ^7^Be and ^212^Pb activity concentration measurements were used in further analysis. The snow depth data was obtained from the closest weather station in Svalbard Lufthavn (SN99843; seklima.met.no), located at the foot of Platåberget. Data from the latter meteorological station was used in case of missing IMS station records.

## Modelling based on atmospheric backward simulations

The operational ATM (Atmospheric Transport Modelling) system used at CTBTO^24,25^ is based on the Lagrangian Particle Dispersion Model FLEXPART^26^, driven by the global meteorological fields provided by the European Centre for Medium-Range Weather Forecasts (ECMWF) and the US National Centers for Environmental Prediction (NCEP).

For the purpose of this study, backward simulations (i.e. from the receptor’s location) were used. A backward simulation is the method of choice when a source is unknown, as it explores the question “where could the air masses seen at the station have come from?”.

To analyse more than 5000 ATM simulations, produced daily between 2008 and 2022, the special software called the WEB-connected GRAPhics Engine (WEB-GRAPE) designed and developed by CTBTO^27^ was used. This software system allows to calculate several ATM products^28^. To reveal the most probable region of influence at ground level (for a selected time period), the functionality allowing for generating network coverage maps was used (for more details please see^29^).

## What can natural radionuclides tell about climate change?

Analysis of the daily measurements of ^7^Be and ^212^Pb is presented in Sects. 4.1. and 4.2, respectively. It is demonstrated that from these two radionuclides, ^212^Pb is the one that may provide evidence of climate change in the case of Spitzbergen.

### Beryllium-7 (^7^Be)

^7^Be (T_1/2_ = 53.3 days) originates from spallation of nitrogen and oxygen nuclei by energetic particles associated with cosmic radiation entering the upper layers of the atmosphere. The concentration of ^7^Be that reaches the surface depends on the ^7^Be production rate which is a function of many parameters, including latitude, altitude and solar activity^30^. ^7^Be attaches predominantly to aerosol particles in the submicron size range^31^ with an activity mean diameter of typically 0.5–0.7 μm^32^. ^7^Be is typically removed from the atmosphere by dry and wet depositions^33^. Figure 2A illustrates that the relation between the concentration of ^7^Be and the precipitation level is inversely proportional. To check if ^7^Be measurements could show any signs of climate change, ^7^Be activity concentration values registered for the month of April during the period 2007-2022 are analyzed (see Figure 2B). April is chosen as the month which is the least influenced by precipitation (see Figure 2A). Figure 2B demonstrates that even if there are yearly variations in the monthly values, no specific trend in the changes can be observed.

However, it should be highlighted that ^7^Be is a valuable indicator of many other processes. For example, the altitude dependency of ^7^Be concentrations combined with its propensity to attach easily to mineral dust makes this radionuclide a well-founded tracer of Saharan dust^34^. In a similar way, ^7^Be particles may attach to volcanic ashes, leading to a local increase in surface ^7^Be concentrations in the area under the influence of subsiding ash plume^35^. Furthermore, ^7^Be can be used as a tracer of global atmospheric circulation patterns that may possibly be caused by global warming. Terzi^36^, for example, apply ^7^Be measurements at IMS radionuclide stations to confirm that major changes in the atmospheric circulation are currently ongoing, including modifications of tropopause heights over the past decade which can at least to some extent be attributable to global warming. Variations of ^7^Be concentrations in air surface layers and their significance in the context of climate change has been studied also by other researchers, for example Jiwen^37^ or Leppänen^38^.

### Lead-212 (^212^Pb)

^212^Pb (T_1/2_ = 10.6 h) is a naturally occurring trace radionuclide of terrestrial origin. It is part of the thorium (4n) decay chain, a primordial radionuclide, and is present in traces in rocks and mineral ores. Its observation throughout the IMS network is consequently directly linked to the local geology. ^212^Pb is released into the atmosphere through radon (^220^Rn in the case of the thorium series), an intermediate decay product of the normal radioactive decay chain. As radon is a noble gas, it is capable of escaping from rocks through cracks and fissures and reach the atmosphere, where it eventually decays into ^212^Pb^39,40^.

**Figure 3:**
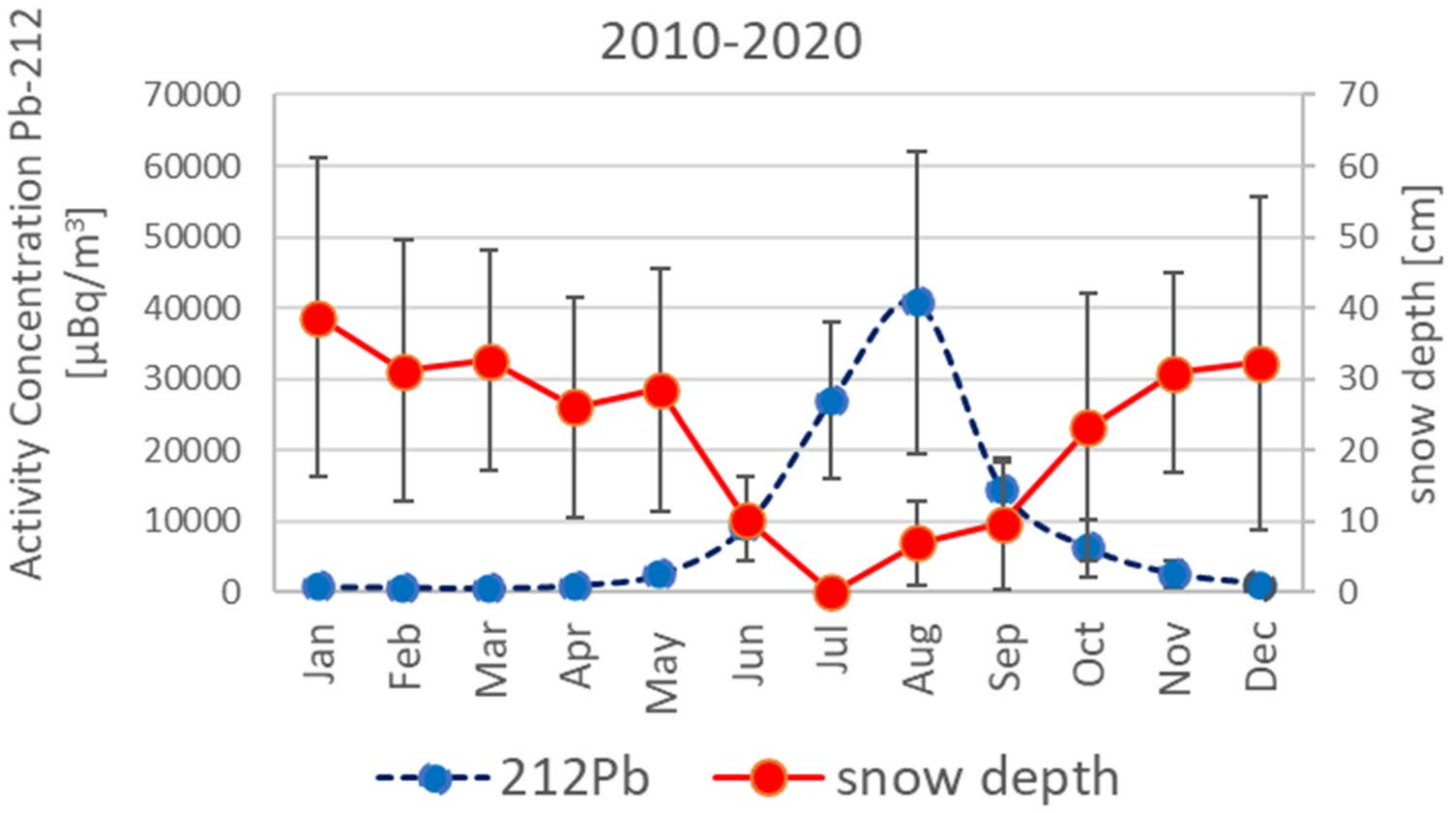
Variations in the monthly average activity concentration of ^212^Pb and the averaged maximum monthly snow depth. The values of snow depth were obtained from the weather station in Svalbard Lufthavn (seklima.met.no), located at the foot of Platåberget.

**Figure 4:**
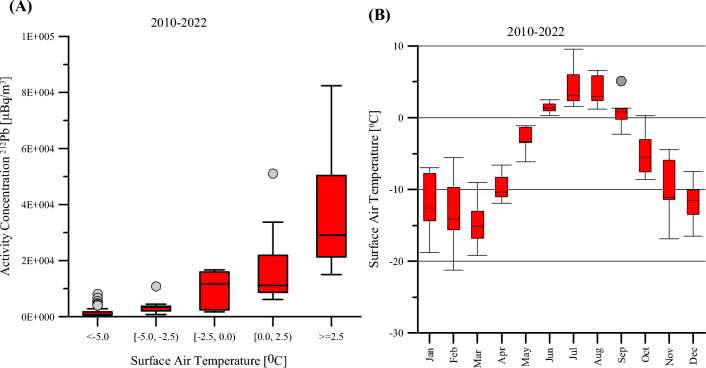
Measurements of ^212^Pb collected at RN49. (**A**) Exponential increase of the ^212^Pb activity concentration with the surface temperature. (**B**) Monthly surface temperature. The caps at the end of each box indicate the extreme values (minimum and maximum), the box is defined by the lower (first) and upper (third) quartiles, and the line in the centre of the box is the median. The dots represent outliers.

In general, it is observed that the measured activity concentration of ^212^Pb increases with the increase of the local ambient temperature and decreases with the increase of wet precipitation in summer and ground snow coverage in winter^41^. Figs 3 and 4 show that similar dependencies are also noticeable through measurements at RN49. Figure 3 demonstrates that the relation between the observed activity concentration of ^212^Pb and the snow depth is inversely proportional. This result is based on the monthly averaged values registered between 2010 and 2022. Figure 4A illustrates that the activity concentration of ^212^Pb increases exponentially with the surface temperature. A similar trend was also observed by Zhang^42^ for a different dataset collected in the Arctic region. This increase is more significant for surface temperatures above zero, i.e. when the snow begins to melt and the ground thaws. An important role is played here by the permafrost (i.e. ground that remains at or below 0^0^C for two or more consecutive years), and more specifically an active layer above the permafrost which thaws during summer and refreeze during winter. The process of refreezing back depends on many factors, for example active-layer moisture content, snow cover timing and thickness, and autumn air temperatures^43^. The longer this process, the shorter is time available for ground cooling, leading to relatively higher ground temperatures. The increased ground temperature facilitates ice melting and ^212^Pb release into the atmosphere through radon. More details on the effect of soil moisture content on radon and thoron exhalation rates can be found for example in Hosoda^44^ or Pyngore^45^. The important role of permafrost and active-layer thickness, shortly outlined above, is thoroughly explained in Christiansen^43^.

Figure 3 and 4B demonstrate that between June and September when the temperature is the highest, the values of ^212^Pb are also significantly higher than during the remaining months which are in general colder and with a higher level of precipitation. It is worth mentioning that in the case of Platåberget on Svalbard, the potential for frost weathering in blockfields is extremely low^46^, therefore it has no influence on the measured ^212^Pb values.

In view of the strong relation between the surface temperature and the activity concentration of ^212^Pb, the question arises whether ^212^Pb measurements could be used as additional source of information about climate change. To verify this hypothesis, a relative difference between the monthly median values of ^212^Pb for the last 7 years (2015–2022) and their baseline values (monthly median values calculated for the period of 15 years (2007–2022)) was calculated. The baseline is based on continuously available data, 2007–2022. For the sake of consistent input dataset size, individual months of data from previous years were considered outside the scope and not included.

**Figure 5:**
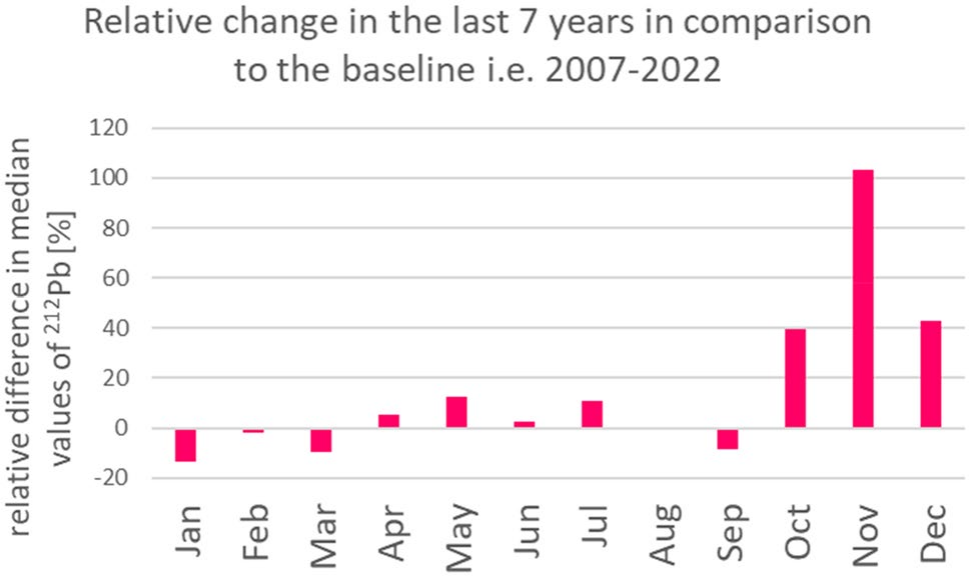
Relative change in monthly median values of ^212^Pb as observed during the last 7 years (2015–2022), in comparison to the baseline calculated for the period: 2007–2022.

Figure 5 demonstrates that the largest relative increase in ^212^Pb values can be observed for the months October, November and December. During the other months the absolute value of the relative difference does not exceed 15%. It is interesting to note that in August there is no observed change.

**Figure 6:**
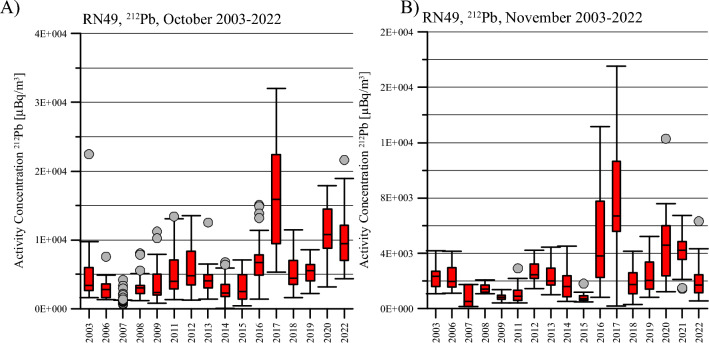
Yearly variations in the monthly values of activity concentrations of ^212^Pb for the station RN49, as observed 2003–2022, for (**A**) October and (**B**) November. The caps at the end of each box indicate the extreme values (minimum and maximum), the box is defined by the lower (first) and upper (third) quartiles, and the line in the centre of the box is the median. The dots represent outliers.

This increasing tendency for the months October and November is also shown in Figs. 6A-B, for the period 2003-2022. The exceptionally high values of ^212^Pb for October 2017 and November 2016 and 2017 suggest much higher values of surface temperature than ever. This finding was also confirmed by the ground-thermal observations in Svalbard conducted by an international team of permafrost researchers^43^. This team investigated ground thermal conditions and active layer thickness at five sites, including one in Endalen (very close to RN49) during the hydrological year 2016–2017 (i.e. 1 September 2016 to 31 August 2017). It was confirmed that this year was particularly warm and wet, delaying the freezing season into mid-November and causing high permafrost temperatures in the top permafrost^43^. This finding was also independently confirmed by Overland^47^ who analyzed the observational record of surface air temperature for Arctic.

**Figure 7:**
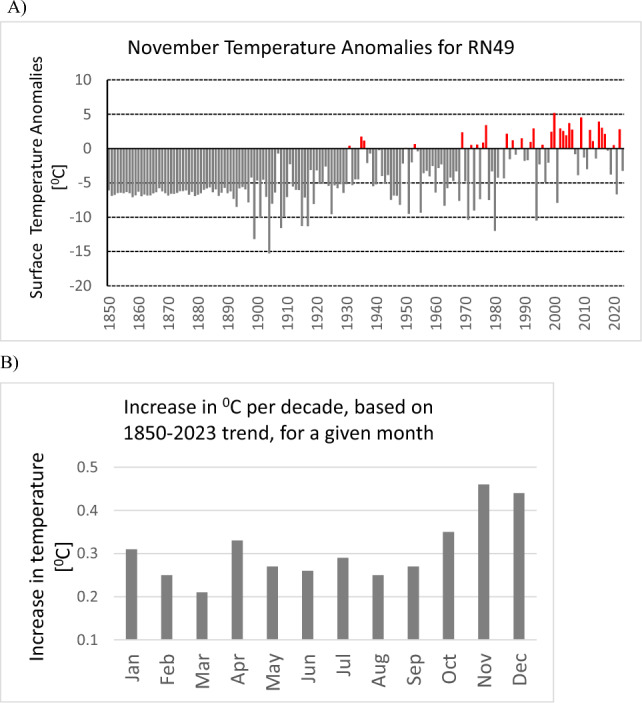
Surface temperature anomalies for the period between 1850 and 2023, for the station RN49, based on data from NOAA National Centers for Environmental Information,(https://www.ncei.noaa.gov/access/monitoring/climate-at-a-glance/global/time-series). (**A**) November temperature anomalies. (**B**) Increase in temperature (per decade) for a given month.

The increasing trend in surface temperature is independently confirmed by time series of monthly temperature anomalies calculated with respect to the 1901–2000 average (see Fig. 7A-B), based on data from NOAA National Centers for Environmental information, Climate at a Glance (https://www.ncei.noaa.gov/access/monitoring/climate-at-a-glance/global/time-series). Figure 7A shows the example of November temperature anomalies for the period between 1850 and 2023. The increase in temperature per decade for November was estimated to be + 0.46 ^°^C. Figure 7B illustrates that such an increase in temperature can be noted also for other months, however the highest values are noted for October, November, and December, what supports our findings based on radionuclide data.

## Potential sources of air masses arriving at the station RN49

Some researchers^20,21^ have described that dust and organic matter deposited on snow and ice may lead to acceleration of snow melting and ultimately to the increase in surface temperature. Knowing that the long-range atmospheric transport will have a very marginal impact on the observed amount of ^212^Pb due to its rather short half lifetime^42,48^, we will investigate the indirect impact of dust (soil originated) aerosols. The recent publication by Meinander^19^ lists newly identified high-latitude dust sources like Alaska, Canada, Denmark, Greenland, Iceland, Svalbard, Sweden and Russia, from which dust particles could easily reach RN49.

**Figure 8:**
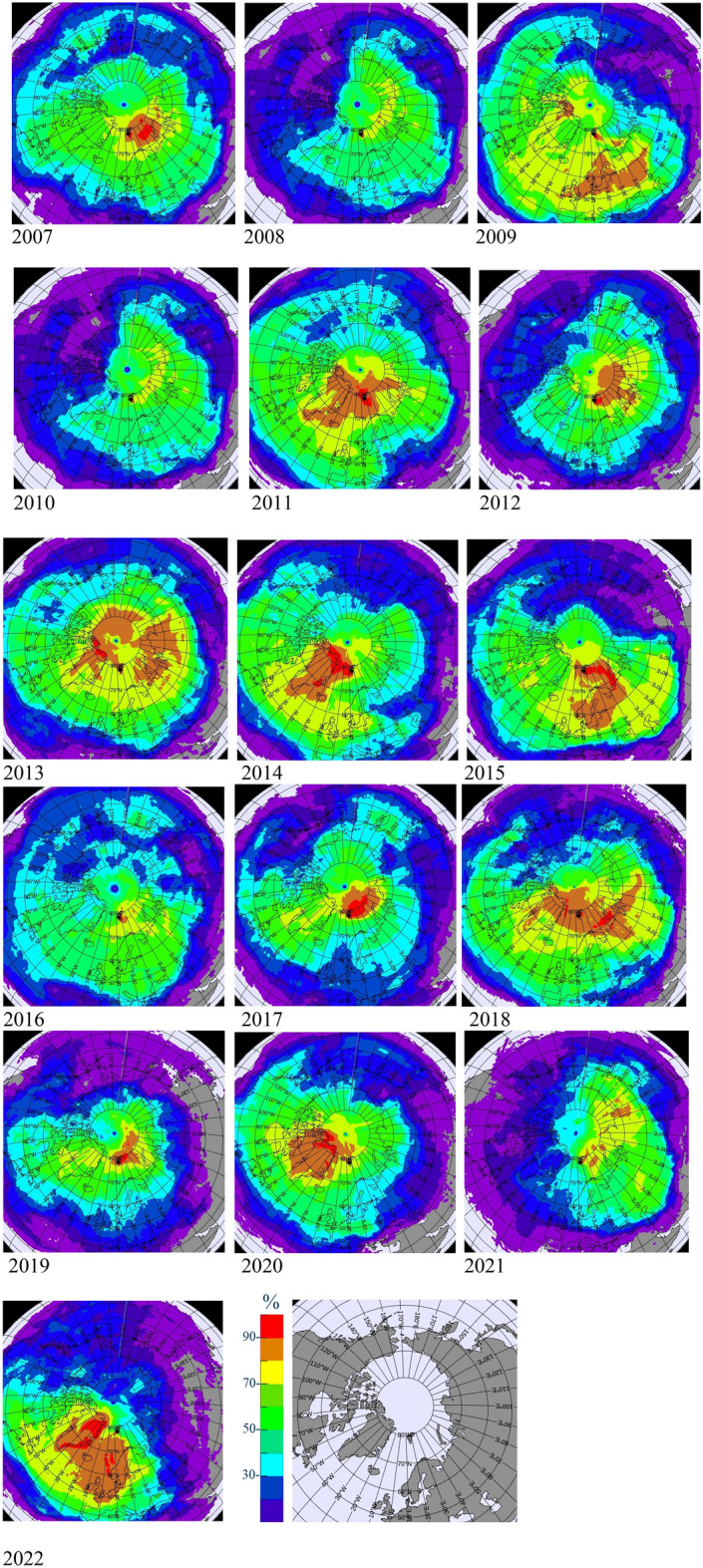
Variability of the wind pattern for the region of Arctic, as demonstrated by the monthly Network Coverage for November, for the period 2007–2022. The orange and red colours (i.e. above 80%) represent the aeras from which the air masses were arriving at RN49 the most frequently during the investigated period. The legend is expressed in %. The last image is added for a reference only. The Network Coverage maps were generated with the CTBTO software, WEB-GRAPE (pls. see Sect. 3 more details).

To check how often the air masses from the above regions arrive at RN49, atmospheric backward simulations produced operationally (14-days long) were used. Since the most significant relative increase in ^212^Pb values is suggested for November, we have selected this month for our complementary study. Following the method described in Sect. 3, the network coverage monthly maps were produced for every month of November of the last 16 years. The results shown in Fig. 8 demonstrate significant interannual variations. When there were strong winds present, the transport time of air masses was much shorter, and a larger area was covered (see Fig. 8, e.g. November 2011). Areas identified as potential sources of dust particles (defined as soil originated particles)^19^, are also highlighted by network coverage maps (with frequency above 80%) as the areas from which the air masses were arriving to RN49 during the investigated period. However, due to the large variability of the wind pattern for the region of Arctic and resulting the most probable region of influence at ground level (expressed by percentage values), it would be difficult to point on one specific region as the one that could potentially explain a connection with the observed increase in surface temperature. However, the results presented in Fig. 8, do not exclude the fact that dust particles from various sources contribute to acceleration of the melting process.

## Discussion

The term "climate change" is very broad. On top of changes in globally averaged surface temperatures, it also encompasses changes in atmospheric circulation, in the size and patterns of natural climate variations, and in local weather^49^.

It is demonstrated here that in the case of Spitzbergen, ^212^Pb may be considered as a pertinent indicator of climate change. It is well known that emanation of uranium decay products from the ground surface is a complex and multifaceted process. Several parameters, such as soil moisture, humidity or porosity are all playing an important role in this complex process. Focus is however given here on the temperature, which is directly measured at the IMS station.

By comparing ^212^Pb monthly median activity concentration values averaged over the period 2008–2022 with their corresponding values over the period 2015–2022, a relative increase (above 40%, see Fig. 5) was noted for the months October, November and December. This can be correlated to a relative increase in surface temperature. However, it should also be noted that during that period (i.e. October to December) the average surface temperature (see Fig. 4 B) is still below 0 ^°^C. It means that an increase in the surface temperature may not bring immediate effects in terms of snow melting.

An increase in surface temperature was reported by many researchers who also proposed their own interpretation of the observed phenomenon. For example, the cloud coverage that is related to the sea surface temperature (as the sea surface is the source of atmospheric water vapor), was indicated as the potential contributing factor^50,51^.

Polar climate change has been often discussed in the context of atmospheric circulation^52^.

A weakening of the polar vortex over the last three or four decades has been reported for example by Garfinkel^53^, Seviour^,^^54^or Kretschmer^55^. Bielec-Bąkowska^56^ studying annual and long-term variability in the occurrence of cyclone and anticyclone centres over Svalbard during the period 1971–2015 showed no major changes in the annual maximum of occurrence of the pressure systems under study. However, regarding a seasonal distribution, they observed a slight predominance of cyclones occurring in November and December, especially towards the end of the multiannual period under study^56^.

Researchers agree that due to large internal variability of the atmospheric circulation, projection of this aspect of climate change is characterized by large uncertainty^57,58^. Both observed and projected changes in the Arctic atmospheric circulation seem to be of insignificant magnitude in comparison to naturally occurring climate variability^52^.

The large internal variability in relation to the wind pattern was also noted in our study (Fig. 8). Based on the analysis of the air mass backward simulations we could demonstrate that air masses arriving at RN49 often come from regions identified as potential sources of dust particles. Soil originated particles (dust) arriving at Spitzbergen, may contribute in a long term to the observed increase in surface temperature.

## Conclusions

This study demonstrates that environmental measurements from the IMS network of the CTBTO can be used to evidence climate change in the case of Spitzbergen.

- It is demonstrated that on an annual basis, with an increase of surface temperature, the concentration of ^212^Pb measured by the station also increases exponentially. As expected, since snow acts as an insulation layer, the higher the snow depth, the lower the measured activity concentration of ^212^Pb.
- Comparison of monthly median values of ^212^Pb for the last 7 years (2015–2022) with a baseline value calculated for the period 2007–2022, indicates a relative increase of ^212^Pb values, for the months October to December.
- The increase in surface temperature (per decade) for October, November and December is also suggested by global temperature anomaly data from NOAA’s Global Surface Temperature Analysis, covering the period 1850 to 2023. This increase is estimated to be + 0.35 ^°^C for October, + 0.46 ^°^C for November and + 0.44 ^°^C for December.
- Air masses arriving at RN49 often come from high-latitude regions recently identified as potential sources of dust (soil originated) particles. However, due to the large variability of the wind pattern in the Arctic region, one specific source cannot be indicated. It is highly possible that particles from various sources contribute to acceleration of the melting process.

This new perspective of data collected by the IMS system may offer a novel and complementary approach to investigating climate change effects. It also demonstrates the potential of IMS data in observing, studying and further investigating the impact of climate change

## Data Availability

Meteorological data were provided by the European Centre for Medium-Range Weather Forecasts under a collaboration agreement with the CTBTO. Data which are not owned by CTBTO were obtained online with data links mentioned in the data set section. The IMS radionuclide data and the SRS files are available directly from the CTBTO upon request by signing a zero-cost vDEC contract **(**which includes a confidentiality agreement**)**. More information on vDEC (the virtual Data Exploitation Centre) is available from the CTBTO website (https://www.ctbto.org/resources/for-researchers-experts/vdec), or by email at vdec@ctbto.org.
